# Organizational readiness for implementing change: a psychometric assessment of a new measure

**DOI:** 10.1186/1748-5908-9-7

**Published:** 2014-01-10

**Authors:** Christopher M Shea, Sara R Jacobs, Denise A Esserman, Kerry Bruce, Bryan J Weiner

**Affiliations:** 1 Department of Health Policy and Management, Gillings School of Global Public Health, University of North Carolina-Chapel Hill, 135 Dauer Drive, CB # 7411, Chapel Hill, NC, USA; 2 Department of Health Policy and Management, University of North Carolina-Chapel Hill, Chapel Hill, NC, USA; 3 Lineberger Comprehensive Cancer Center, University of North Carolina-Chapel Hill, Chapel Hill, NC, USA; 4 Department of Medicine, Division of General Medicine and Clinical Epidemiology, School of Medicine, University of North Carolina-Chapel Hill, Chapel Hill, NC, USA; 5 Department of Biostatistics, Gillings School of Global Public Health, University of North Carolina-Chapel Hill, Chapel Hill, NC, USA; 6 Pact Inc, Washington, DC, USA

**Keywords:** Readiness for change, Measure development, Psychometrics

## Abstract

**Background:**

Organizational readiness for change in healthcare settings is an important factor in successful implementation of new policies, programs, and practices. However, research on the topic is hindered by the absence of a brief, reliable, and valid measure. Until such a measure is developed, we cannot advance scientific knowledge about readiness or provide evidence-based guidance to organizational leaders about how to increase readiness. This article presents results of a psychometric assessment of a new measure called Organizational Readiness for Implementing Change (ORIC), which we developed based on Weiner’s theory of organizational readiness for change.

**Methods:**

We conducted four studies to assess the psychometric properties of ORIC. In study one, we assessed the content adequacy of the new measure using quantitative methods. In study two, we examined the measure’s factor structure and reliability in a laboratory simulation. In study three, we assessed the reliability and validity of an organization-level measure of readiness based on aggregated individual-level data from study two. In study four, we conducted a small field study utilizing the same analytic methods as in study three.

**Results:**

Content adequacy assessment indicated that the items developed to measure change commitment and change efficacy reflected the theoretical content of these two facets of organizational readiness and distinguished the facets from hypothesized determinants of readiness. Exploratory and confirmatory factor analysis in the lab and field studies revealed two correlated factors, as expected, with good model fit and high item loadings. Reliability analysis in the lab and field studies showed high inter-item consistency for the resulting individual-level scales for change commitment and change efficacy. Inter-rater reliability and inter-rater agreement statistics supported the aggregation of individual level readiness perceptions to the organizational level of analysis.

**Conclusions:**

This article provides evidence in support of the ORIC measure. We believe this measure will enable testing of theories about determinants and consequences of organizational readiness and, ultimately, assist healthcare leaders to reduce the number of health organization change efforts that do not achieve desired benefits. Although ORIC shows promise, further assessment is needed to test for convergent, discriminant, and predictive validity.

## Introduction

Attempts to implement new programs, practices, or policies in organizations often fail because leaders do not establish sufficient organizational readiness for change [1]. Organizational readiness refers to ‘the extent to which organizational members are psychologically and behaviorally prepared to implement organizational change’ [2]. When organizational readiness is high, members are more likely to initiate change, exert greater effort, exhibit greater persistence, and display more cooperative behavior, which overall results in more effective implementation of the proposed change [3]. Conversely, when organizational readiness is low, members are more likely to view the change as undesirable and subsequently avoid, or even resist, planning for the effort and participating in the change process.

Although organizational readiness for change in healthcare settings has been identified as an important issue [4], research on the topic is hindered by the absence of a brief, reliable, and valid measure of the construct. Until recently, the primary focus in the literature has been on individual readiness for change, not on organizational readiness for change [2]. Although there have been several attempts at measuring organizational readiness (*e.g.*, [5-7]), most available instruments are not theory-based and exhibit limited reliability and validity [2,6]. Furthermore, those with desirable psychometric properties have too many items to be practical for use in busy healthcare settings [4]. Until a brief, reliable, and valid measure is developed, we cannot advance scientific knowledge of the determinants or outcomes of readiness or provide evidence-based guidance to organizational leaders about how to increase readiness.

In this article, we report the results of a psychometric assessment of a new, theory-based measure we call Organizational Readiness for Implementing Change (ORIC). We developed ORIC by drawing on Weiner’s theory of organizational readiness for change [8] and assessed its content adequacy, structural validity, reliability, and construct validity in a series of studies. These studies provide psychometric evidence for a brief yet robust measure that could be used to advance implementation science and practice.

## Conceptual framework

Organizational readiness for change is a multilevel construct that can be assessed at the individual or supra-individual levels (*e.g.*, team, department, or organization). In this analysis, we focus on the supra-individual level because the implementation of many promising innovations in healthcare, such as patient-centered medical homes, Accountable Care Organizations, and electronic health records, require collective, coordinated actions by many organizational members [8]. There are three considerations that researchers should keep in mind when measuring readiness for change at supra-individual levels. First, items should be group-referenced (*e.g.*, ‘We are ready to…’) rather than self-referenced (*e.g.*, ‘I am ready to…’) so that they focus respondents’ attention on collective readiness rather than personal readiness [8]. Second, assessment should involve multiple respondents from the same unit. Proxy reporting of collective readiness by single respondents (*e.g.*, the CEO) is unlikely to generate valid data [2]. Third, inter-rater agreement should be checked before aggregating individuals’ readiness perceptions to supra-individual levels of analysis. If, for example, one-half of an organization’s members perceive readiness to be high and one-half perceive it to be low, then the average of organizational members’ perceptions of readiness describes none of their views [8].

**Figure 1:**
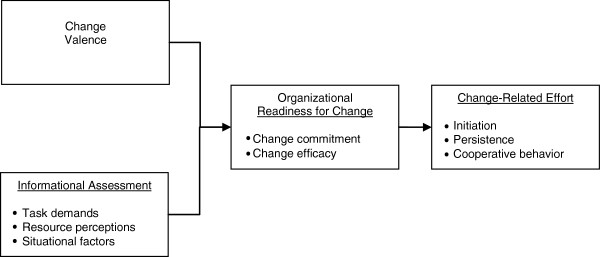
**Determinants and outcomes of organizational readiness for change.** *Adapted from Weiner, B.J., A theory of organizational readiness for change*. Implement Sci*, 2009. 4: p. 67.

Organizational readiness for change is not only a multilevel construct, but a multi-faceted one. We used Weiner’s theory of organizational readiness for change [8] to identify and define the two facets we aimed to measure as well as the immediate determinants of each (Figure 1). The first facet of readiness, change commitment, reflects organizational members’ shared resolve to implement a change. A hypothesized determinant of change commitment is change valence. Organizational members may value an organizational change for any number of reasons; why they value it may be less important than how much they value it. The second facet of readiness, change efficacy, reflects organizational members’ shared belief in their collective capability to implement a change [3,8]. Hypothesized determinants of change efficacy include task knowledge, resource availability, and situational factors. Change efficacy is expected to by high when organizational members know what to do and how to do it, when they perceive they have the resources they need to implement the change, and when they perceive situational factors such as timing to be favorable. An immediate outcome of readiness is organizational members’ change-related effort. For example, when readiness is high, organizational members are more likely to initiate the change, put forth greater effort in support of the change, and exhibit greater persistence in the face of obstacles.

Given the potential impact of organizational readiness for change on implementation outcomes, a valid, theory-based measure would be useful for research and practice. The measure should reflect both facets of readiness—change commitment and change efficacy—and differentiate the facets from their determinants to ensure direct measurement of the readiness construct [9]. In addition to being valid and reliable, the assessment must be brief in order to be practical in busy healthcare settings.

## Methods

We conducted four studies to assess the psychometric properties of a newly developed, theory-based measure of organizational readiness for implementing change (ORIC). In study one, we assessed the content adequacy of the new measure using quantitative methods [10]. In study two, we examined the measure’s factor structure and reliability in a laboratory simulation. In study three, we assessed the reliability and validity of an organization-level measure of readiness based on aggregated individual-level data from study two. In study four, we conducted a small field study to validate the results of study three.

## Study one

### Method

Content adequacy refers to ‘the degree to which a measure’s items are a proper sample of the theoretical content domain of a construct’ [10]. Typically, researchers assess content adequacy by asking a small group of experts to assess whether a measure’s items seem representative of the construct’s theoretical content. Often, the assessment is informal and qualitative. In this study, we took a formal, quantitative approach by asking a large group of judges to rate the extent to which items reflect the constructs they were intended to measure.

### Design, participants, and procedures

Study participants were a convenience sample of 98 students enrolled in undergraduate, masters, and doctoral programs in health policy and management at a university located in the southeastern United States. Convenience samples are acceptable for content adequacy studies because such studies require no particular expertise or experience, merely sufficient reading skill and intellectual ability to perform the rating task [10]. Twenty-eight percent of study participants were men, 72% women. Fourteen percent were 18 – 21 years old, 19% were 22 – 25 were years old, 18% were 26 – 28 years old, 7% were 29 – 31 years old, and 33% were 32 years old or older.

Study participants were randomly assigned to complete one of two web-based surveys. One survey consisted of 15 items that reflected two related constructs: change commitment and change valence. The other survey consisted of 15 items that reflected three related constructs: change efficacy, task knowledge, and resource availability. Each survey provided detailed instructions and examples of how to perform the rating task. Using the definitions that we provided, participants rated the extent to which they thought each item reflected each construct in the survey. For example, participants receiving the first survey rated each item twice: once to indicate the extent to which they thought the item reflected change commitment, and again to indicate the extent to which they thought the item reflected change valence. Rating was done using a five-point ordinal scale in which a ‘1’ indicated that an item ‘does not reflect the concept at all’ and a ‘5’ indicates that an item ‘reflects the concept well’.

To minimize ordering effects, one-half of the participants receiving the first survey were randomly assigned to perform the change commitment rating task first; the other one-half performed the change valence rating task first. To minimize learning effects, items were presented in random order within each rating task. The same procedure was used to minimize ordering effects and learning effects in the second survey.

### Measures

Five items in the first survey were written to measure change commitment (*e.g.*, ‘we are committed to implementing this change’). Ten items were written to measure some aspect of change valence: perceived need (*e.g.*, ‘we need to implement this change’), perceived benefit (*e.g.*, ‘we believe this change will make things better’), perceived timeliness (*e.g.*, ‘we see this change as timely’), and perceived compatibility (*e.g.*, ‘we feel this change is compatible with our values’). We also included a ‘distracter’ item to check on participants’ attention and diligence in performing the rating task (*e.g.*, ‘we know what it takes to implement this change’).

Six items in the second survey were written to measure change efficacy (*e.g.*, ‘we can coordinate tasks so that implementation goes smoothly’). Four items were written to measure task knowledge (*e.g.*, ‘we know what we need to do to implement this change’). Five items were written to measure resource availability (*e.g.*, we have the resources we need to implement this change’). We also included a ‘distracter’ item to check on participants’ attention and diligence in performing the rating task (*e.g.*, ‘the timing is good for implementing this change’).

### Analysis

Content adequacy involves judgments of item content in relation to theoretically-defined constructs [9]. We considered the item adequate if it met three conditions: the item’s highest mean corresponded to the intended aspect of organizational readiness for implementation (*e.g.*, change commitment and not change valence) [11]; the item’s mean on the intended aspect of organizational readiness for implementation was at least 0.20 units higher than its mean on its hypothesized determinants [11]; and the item’s mean must be greater than or equal to 4 on its intended aspect of organizational readiness [11]. In addition, we performed an analysis of variance (ANOVA) to compare the item’s mean rating on the hypothesized theoretical construct (*e.g.*, change commitment) to the item’s ratings on alternative constructs (*e.g.*, change valence) [12].

## Results

In Table 1, the first five items (C1 – C5) were intended to measure change commitment. The first four of these items (C1 – C4) satisfied all three conditions of the content adequacy test. The fifth item (C5) did not satisfy any condition of the content adequacy test. Study participants rated this item as equally reflecting the concepts of change commitment and change valence. In the absence of more semantic context, the verb ‘want’ could connote either motivation (commitment) or desire (value) to implement change. Although this item did not pass the content adequacy test, we retained it for further testing in circumstances where the semantic context was more clearly defined. None of the items intended to measure change valence (V1 – V10) passed the content adequacy test as measures of change commitment. This suggests that study participants were able to distinguish items intended to measure change commitment from items intended to measure its hypothesized determinant. Also, the distracter item (D1) did not satisfy the three conditions for fit with the construct of change commitment or change valence.

**Table 1:** Content adequacy assessment of items intended to measure the change commitment dimension of organizational readiness for implementation (study one)

	**Mean rating for commitment**	**Mean rating for valence**	**Pass condition 1?**	**Pass condition 2?**	**Pass condition 3?**
C1. We are committed to implementing this change.	4.78*	2.52	Yes	Yes	Yes
C2. We are determined to implement this change.	4.64*	2.28	Yes	Yes	Yes
C3. We are motivated to implement this change.	4.26*	2.79	Yes	Yes	Yes
C4. We will do whatever it takes to implement this change.	4.73*	2.41	Yes	Yes	Yes
C5. We want to implement this change.	3.47	3.32	No	No	No
V1. We feel this change is compatible with our values.	2.23	4.37*	Yes	Yes	Yes
V2. We need to implement this change.	3.10	3.6	Yes	No	No
V3. We believe this change will benefit our community.	2.13	4.28*	Yes	Yes	Yes
V4. We believe it is necessary to make this change.	2.91	3.78*	Yes	No	No
V5. We believe this change will work.	2.41	3.34*	Yes	No	No
V6. We see this change as timely.	2.21	3.37*	Yes	No	No
V7. We believe this change is cost-effective.	2.23	3.06*	Yes	No	No
V8. We believe this change will make things better.	2.30	4.47*	Yes	Yes	Yes
V9. We feel that implementing this change is a good idea.	2.54	4.14*	Yes	Yes	Yes
V10. We value this change.	2.30	4.73*	Yes	Yes	Yes
D1. We know what it takes to implement this change.	3.10*	1.93	N/R	N/R	N/R

*Denotes statistically significant differences (p < 0.05) for one-way ANOVA between mean ratings for Commitment and Valence.

*Notes:* The first five items were intended to measure change commitment (C). The last item was a distractor (D). The remaining items were intended to measure aspects of change valence (V). The following definitions were provided to participants: *Change commitment* refers to organizational members’ shared resolve to pursue courses of action that will lead to the successful implementation of the change effort. *Change valence* refers to the value that organizational members assign to a specific, impending organizational change. For example, do they think the change is needed, important, beneficial, or worthwhile?

In Table 2, the first six items (E1 – E6) were intended to measure change efficacy. All six items satisfied all three conditions of the content adequacy test. None of the items intended to measure task knowledge (K1 – K4) or resource availability (A1 – A5) passed the content adequacy test as measures of change efficacy. This finding suggests that study participants were able to distinguish items intended to measure change efficacy from items intended to measure its hypothesized determinants. Finally, the distracter item (D2) did not satisfy the three conditions for fit with change efficacy, task knowledge or resource availability.

**Table 2:** Content adequacy assessment of items intended to measure the change efficacy dimension of organizational readiness for implementation (study one)

	**Mean rating for efficacy**	**Mean rating for task knowledge**	**Mean rating for resource availability**	**Pass condition 1?**	**Pass condition 2?**	**Pass condition 3?**
E1. We can keep the momentum going in implementing this change.	4.40*	1.80	1.56	Yes	Yes	Yes
E2. We can manage the politics of implementing this change.	4.24*	1.84	1.66	Yes	Yes	Yes
E3. We can support people as they adjust to this change.	4.28*	1.94	2.16	Yes	Yes	Yes
E4. We can get people invested in implementing this change.	4.42*	1.96	1.78	Yes	Yes	Yes
E5. We can coordinate tasks so that implementation goes smoothly.	4.34*	2.34	1.58	Yes	Yes	Yes
E6. We can keep track of progress in implementing this change.	3.54*	2.18	1.54	Yes	Yes	Yes
K1. We know how much time it will take to implement this change.	1.69	4.64*	1.62	Yes	Yes	Yes
K2. We know how much time it will take to implement this change.	1.71	4.82*	1.64	Yes	Yes	Yes
K3. We know what resources we need to implement this change.	1.77	4.62*	2.32	Yes	Yes	Yes
K4. We know what each of us has to do to implement this change.	2.32	4.80*	1.64	Yes	Yes	Yes
A1. We have the equipment we need to implement this change.	2.00	2.30	4.88*	Yes	Yes	Yes
A2. We have the expertise to implement this change.	2.80	2.94	4.48*	Yes	Yes	Yes
A3. We have the time we need to implement this change.	2.59	2.54	4.52*	Yes	Yes	Yes
A4. We have the skills to implement this change.	2.95	3.02	4.37*	Yes	Yes	Yes
A5. We have the resources we need implement this change.	1.91	2.38	4.85*	Yes	Yes	Yes
D2. The timing is good for implementing this change.	2.55	1.76	2.00	N/R	N/R	N/R

*Denotes statistically significant differences (p < 0.05) for ANOVA between mean ratings for Efficacy, Task Knowledge, and Resource Availability.

*Notes:* The first six items were intended to measure change efficacy (E). The next four items were intended to measure task knowledge (K). The next five items were intended to measure resource availability (A). The last item was a distractor (D). The following definitions were provided to participants: *Change efficacy* refers to organizational members’ shared beliefs (or confidence) in their collective capabilities to organize and execute the courses of action required to implement the change successfully. *Task knowledge* refers to organizational members’ *knowledge* of the tasks that have to be performed, the resources that will be needed, the amount of effort that will be required, and the amount of time that it will take to implement a change. *Resource availability* refers to organizational members’ perceptions of the *availability* of money, people, equipment, and other resources needed to implement a change.

Table 1 and Table 2 note statistically significant differences identified by the ANOVA procedures comparing each item’s mean rating on its hypothesized theoretical construct and its ratings on alternative constructs.

## Study two

### Method

In study two, we ascertained the factor structure and reliability of our new measure in a laboratory study wherein we manipulated the two aspects of organizational readiness in vignettes that described a hospital about to implement a major organizational change.

### Design, participants, and procedures

The study used a 2 (high- versus low-change commitment) × 2 (high- versus low-change efficacy) between-subjects design. Study participants read one of four randomly assigned vignettes that described a hospital’s readiness to implement meaningful use of electronic health records. They then rated the hospital’s readiness for implementation as they believed a hospital employee would rate it. Hospital readiness was manipulated in the vignettes by describing various levels of the following: employee knowledge of meaningful use, employee morale and enthusiasm, resources available for implementation, and situational factors such as leadership stability and timing of the implementation. Study participants were a convenience sample of 140 students enrolled in undergraduate, masters, and doctoral programs in health policy and management or health behavior at a university located in the Southeastern United States. These programs familiarize students with the structures, workforce, and services of healthcare organizations. Twenty-six percent of study participants were men; 74% were women. Eleven percent were 18 – 21 years old, 25% were 22 – 25 years old, 28% were 26 – 28 years old, 16% were 29 – 31, and 20% were 32 years old or older. A convenience sample was acceptable because participants needed only sufficient intellectual capability to comprehend the vignette and perform the rating task. This approach has been used in previous studies [13].

### Measures

Participants rated the hospital’s readiness for implementation on 12 items using a 5-point ordinal scale that ranged from ‘disagree’ to ‘agree’ (Additional file 1 contains the items). All five items used to assess change commitment in study one were included. Although one of the change commitment items did not pass the content adequacy tests (‘We want to implement this change’), we retained it in order to see how it performed in a more clearly defined semantic context (*i.e.*, the vignettes). All six items used to assess change efficacy in study one were also included. A seventh change efficacy item was added (‘We can handle the challenges that might arise in implementing this change’) in order to capture the organization’s ability to respond to problems that emerge during implementation. Although not included in study one, this item passed a content adequacy test involving the same methods but with only 10 study participants. Item content did not change from study one to study two, although item wording did. In study two, efficacy items were phrased ‘People who work here are confident that…’ This wording was not used in study one because it would have made it obvious to raters that they were efficacy items (based on the use the word ‘confident’), which would have defeated the purpose of the content adequacy test.

### Analysis

To determine whether change commitment and change efficacy are distinct, yet related facets, we conducted an exploratory principal-axis factor analysis (EFA) with oblique rotation, followed by a confirmatory factor analysis (CFA) specifying two factors. We chose oblique rotation because we expected, based on theory [9], that change commitment and change efficacy represented interrelated facets of organizational readiness and, therefore, would be correlated. Oblique rotation allows for identification of unique contributions to variance and excludes contributions to variance from overlap between the correlated factors [14]. To determine the number of factors to retain in the EFA, we ran the parallel analysis procedures (PA), which Zwick and Velicer [15] found outperforms other methods such as the Kaiser criterion, scree plot, and Bartlett’s chi-square test. In PA, a set of random data correlation matrices are created and their eigenvalues are computed. These eigenvalues are compared to those computed from the study data, and only those factors from the study data with eigenvalues greater than those from the random data are retained. To interpret the retained factors and assess item performance, we used the following conventions: factor loadings greater than 0.6, factor cross-loadings less than 0.3, communalities greater than 0.5, and face-valid factor membership [16]. To assess CFA model fit, we used the following rules of thumb: comparative fit index (CFI) and Tucker-Lewis fit index (TLI) equal to or greater than 0.95, standard root mean square residual (SRMR) less than 0.05, and root mean square error of approximation (RMSEA) equal to or less than 0.06 [17].

We then formed scales for change commitment and change efficacy based on the CFA results and computed alpha coefficients for each scale to assess inter-item consistency. Finally, we conducted a 2 × 2 analysis of variance (ANOVA) to determine whether change commitment and change efficacy varied as expected by the manipulation of information in the vignettes.

## Results

EFA yielded two factors with eigenvalues greater than 1 and greater than those generated from the PA of 10 random data correlation matrices. All five items intended to measure change commitment (C1 – C5) exhibited factor loadings greater than 0.6 on the first retained factor, cross-loadings less than 0.25 on the second factor, and communalities greater than 0.5 (see Table 3). Five of the seven items intended to measure change efficacy exhibited factor loadings greater than 0.6 on the second retained factor, cross-loadings less than 0.25 on the first factor, and communalities greater than 0.5. Based on these results, we interpreted the factors as Change Commitment (factor one) and Change Efficacy (factor two). Two items intended to measure change efficacy exhibited loadings between 0.5 and 0.6 on the Change Commitment factor and cross-loadings on the Change Efficacy factor less than 0.25. These items were: (1) ‘People who work here confident that they can keep the momentum going in implementing this change’, and (2) ‘People who work here feel confident that the organization can get people invested in implementing this change’. Apparently, these items had a motivational connotation that study participants associated with items intended to measure change commitment. Since these two items did not load on the expected factor (and lacked face validity as measures of change commitment), we dropped them and re-ran the EFA. Re-analysis did not alter the number of factors or the pattern of factor loadings for the remaining items. We then ran a two-factor CFA using the ten items retained from the re-analysis. The two-factor CFA converged and demonstrated a strong fit when change commitment and change efficacy were allowed to correlate. The comparative fit index (CFI) equaled 0.98, the Tucker-Lewis fit index (TLI) = 0.97, the standard root mean square residual (SRMR) was 0.04 and the root mean square error of approximation (RMSEA) was = 0.06. Standardized parameter estimates are provided in Table 4. No *post hoc* modifications were necessary given the good fit indicated by the fit indices; residual analysis did not indicate any problems.

**Table 3:** Exploratory and confirmatory factor analysis of organizational readiness for implementation items (study two)

			**EFA factor loadings**		CFA* standardized factor loadings	
	**Mean**	**Standard deviation**	**I**	**II**	**I**	**II**
C1. Are committed to implementing this change	3.07	1.33	**0.80**	0.07	0.872 (0.025)	
C2. Are determined to implement this change	2.82	1.29	**0.85**	0.04	0.898 (0.021)	
C3. Are motivated to implement this change	2.87	1.45	**0.92**	−0.04	0.874 (0.024)	
C4. Will do whatever it takes to implement this change	2.58	1.20	**0.68**	0.10	0.784 (0.036)	
C5. Want to implement this change	3.29	1.32	**0.77**	−0.07	0.769 (0.038)	
E1. Feel confident that they can keep the momentum going in implementing this change	2.53	1.22	**0.55**	0.16	--	--
E2. Feel confident that they can manage the politics of implementing this change	2.78	1.07	0.01	**0.73**		0.763 (0.042)
E3. Feel confident that the organization can support people as they adjust to this change	2.77	1.29	0.16	**0.66**		0.800 (0.038)
E4. Feel confident that the organization can get people invested in implementing this change	2.92	1.46	**0.58**	0.10	--	--
E5. Feel confident that they can coordinate tasks so that implementation goes smoothly	2.71	1.16	0.02	**0.84**		0.768 (0.041)
E6. Feel confident that they can keep track of progress in implementing this change	2.97	1.15	0.09	**0.63**		0.684 (0.051)
E7. Feel confident that they can handle the challenges might arise in implementing this change	2.91	1.29	−0.03	**0.79**		0.838 (0.033)

*Notes:* All items begin ‘People who work here…’. Bold EFA results indicate the highest factor loading for each item.

*Standardized factor loadings for two factor confirmatory factor analysis; Standard Error in parenthesis.

for CFA: CFI = 0.981; TLI = 0.975; SRMR = 0.042; RMSEA = 0.06.

**Table 4:** Analysis of variance summary for vignette hospitals (study two)

		**Change**		**Change**	
		**Commitment**		**Efficacy**	
**Source**	***df***	***F***	**Η**	***F***	**η**
CC	1	50.29***	0.24	1.15	0.01
CE	1	0.00	0.00	23.48***	0.14
CC X CE	1	24.64***	0.12	6.87**	0.04
Residual	137				

*Notes:* The dependent variables are the Change Commitment and Change Efficacy Scales.

CC = change commitment factor (high versus low, manipulated in vignettes).CE = change efficacy factor (high versus low, manipulated in vignettes).

Eta-squared describes the ratio of variance explained in the dependent variable by a predictor while controlling for other predictors.

**p <0.01.

***p <0.001.

Alpha coefficients for the five-item Change Commitment Scale and the five-item Change Efficacy Scale were 0.92 and 0.88 respectively. The correlation between the unit-weighted scale scores was similar to that between the factors (*r* = 0 0.56, *p* <0.001).

The 2 × 2 ANOVAs revealed small- to medium-size main effects for each manipulation in the vignette and small-size interaction effects (see Table 4) [18]. The manipulation of information about change commitment and change efficacy explained 36% of the variation in the Change Commitment Scale scores and 19% of the Change Efficacy Scale scores. The variance accounted for seemed reasonable given that study participants had to infer the level of change commitment and change efficacy in the vignettes. Plots of the marginal means (not shown) indicate that participants had no difficulty distinguishing change commitment and change efficacy when these factors, individually or jointly, were low. They had more difficulty distinguishing them when both factors were high.

## Study three

### Method

Having established, at least provisionally, the reliability of our new measure at the individual level of analysis, we sought next to ascertain its reliability and validity at the organization level of analysis. Organizational readiness for implementing change is one of many constructs that are potentially relevant to implementation science that can be conceptualized at the organizational level of analysis even though the source of data for the construct resides at the individual level. Although it is tempting to simply compute an organization-level mean from the individual-level data, it is important to first check the reliability and validity of that mean to determine whether it is an adequate representation of the organization-level construct [19,20]. Organizational readiness is conceived as a ‘shared team property’, that is, a psychological state that organizational members hold in common [8]. Whether this shared team property exists in any given organization is an empirical issue that requires examination of inter-rater reliability and inter-rater agreement. If sufficient inter-rater reliability and inter-rater agreement exist (*i.e.*, organizational members agree in their readiness perceptions), then analysis of organizational readiness as a shared team property can proceed. That is, an organization-level mean can be computed that reliably and validly reflects the perceptions of organizational members as a group. If insufficient inter-rater reliability and inter-rater agreement exist (*i.e.*, organizational members disagree in their readiness perceptions), organizational readiness as a shared team property does not exist. In that case, an organization-level mean does not reliable and validly reflect the perceptions of organizational members as a group. In study three, we assess whether change commitment and change efficacy can be measured reliably and validly at the organizational level of analysis using individual-level data from study two.

### Design, participants, and procedure

Study three involved the same design, participants, and procedures as study two. Thus, the data were the same, but they were analyzed differently. In study three, we treated the 140 study participants as if they were employees of the hospitals depicted in the vignettes. Thus, for each of the four hospitals, there were 35 ‘employees’ rating the hospital’s readiness to implement meaningful use of electronic health records. One of the advantages of using data from a laboratory study is that we can test whether our measures reliably and validly differentiate organizations that systematically differ by design in levels of change commitment and change efficacy.

### Measures

Study three used the five-item Change Commitment Scale and the five-item Change Efficacy Scale developed in study two.

### Analysis

To assess the reliability of the organization-level means for change commitment and change efficacy, we computed values for two intraclass correlation coefficients—ICC(1) and ICC(2)—from a one-way random-effects ANOVA. ICC(1) provides an estimate of the extent to which individual-level variability on a given measure is explained by higher level units [20,21]. ICC(1) can also be interpreted as an estimate of the extent to which raters are interchangeable—that is, the extent to which one rater in a group could represent all raters within that group [20]. The larger the value of ICC(1), the more alike the raters are. ICC(2) is a mathematical function of ICC(1), adjusted for group size. ICC(2) indicates the reliability or stability of group-level means in a sample [22]. The larger the value of ICC(1) and the larger the number of respondents per group, the more reliable the group means and, hence, the greater the value of ICC(2).

To assess the validity of the organization-level means as measures of organization-level constructs, we computed and tested the statistical significance of two indices of inter-rater agreement: *r*_*WG(J)*_ and *AD*_*M(J)*_. The *r*_*WG(J)*_ index indicates the extent of consensus, agreement, or within-unit variability in a multi-item scale by comparing within-group variances to an expected variance under the null hypothesis of no agreement [20]. We assumed the null followed a uniform (rectangular) distribution. The *AD*_*M(J)*_ index, also known as the mean absolute deviation, is used less often than the *r*_*WG(J)*_, but allows more direct conceptualizations of inter-rater agreement in the units of the original measure. Both the indices and their respective critical values, which were obtained from an empirical distribution based on 100,000 simulations and corresponded to a 0.05 level of statistical significance, were calculated using the Multilevel Modeling in R package [23,24]. The *r*_*WG(J)*_ critical value is calculated based on the 95th percentile and the *AD*_*M(J)*_ based on the 5^th^ percentile [23]. Formulae for *r*_*WG(J)*_, *AD*_*M(J),*_ ICC(1), and ICC(2), can be found in the Appendix.

## Results

The one-way ANOVA for the Change Commitment Scale yielded an ICC(1) of 0.72, and an ICC(2) of 0.98 (p <0.001). The one-way ANOVA for the Change Efficacy Scale yielded an ICC(1) of 0.51, and an ICC(2) of 0.97 (p <0.001). The magnitude and statistical significance of these correlations indicate the organization-level means for the Change Commitment scale and the Change Efficacy scale were reliable. More individual-level variance was explained by hospital (vignette) assignment for change commitment than for change efficacy (72% versus 51%).

For the Change Commitment Scale, the sample values for *r*_*WG(J)*_ and *AD*_*M(J)*_ for the ensemble of four hospitals were 0.87 and 0.73 respectively. For the Change Efficacy Scale, the sample values for *r*_*WG(J)*_ and *AD*_*M(J)*_ for the ensemble of four hospitals were 0.82 and 0.80 respectively. Table 5 summarizes the empirical distributions in terms of their means, medians, standard deviations, 5^th^ percentiles, and 95^th^ percentiles. For both scales, the sample values for the ensemble of hospitals for *r*_*WG(J)*_ exceeded the 95^th^ percentile of their corresponding null distributions and the corresponding p-values were almost zero. Likewise, the sample values for *AD*_*M(J)*_ for the ensemble of hospitals were smaller than the 5^th^ percentile of their corresponding null distributions and the corresponding p-values were almost zero. Therefore, for both the *r*_*WG(J)*_ and *AD*_*M(J)*_ indexes, the null hypothesis of no agreement in the ensemble of hospitals in the sample was rejected.

**Table 5:** **Significance tests for the means of *r***_***WG(J) ***_**and *AD***_***M(J) ***_**for vignette hospitals as a group (study three)**

	**Distribution based on simulation**						
**Variable**	**Mean**	**Median**	**Standard deviation**	**5**^**th **^**percentile**	**95**^**th **^**percentile**	**Sample values**	***p *** **value**
Change commitment (5 items)							
*AD*_*M(J)*_ mean	1.20	1.20	0.03	1.15	1.25	0.73	0.00
*r*_*WG(J)*_ mean	0.11	0.11	0.07	0.00	0.25	0.87	0.00
Change Efficacy (5 items)							
*AD*_*M(J)*_ mean	1.20	1.20	0.03	1.15	1.26	0.80	0.00
*r*_*WG(J)*_ mean	0.12	0.11	0.08	0.00	0.25	0.82	0.00

*Note*: For each of the four statistics (*AD*_*M(J)*_ mean and *r*_*WG(J)*_ mean for each scale), we obtained an empirical distribution based on 100,000 simulated random samples. The distributions are summarized in terms of their means, medians, standard deviations, and 5^th^ and 95^th^ percentiles. Consider the *r*_*WG(J)*_ mean for the five-item Change Commitment scale. Its sample value was 0.87. Simulations under the uniform (rectangular) null distribution indicate that the empirical distribution has a mean of 0.11 and a 95^th^ percentile of 0.25, which is much lower than the observed value of 0.87. Thus, the corresponding *p*-value is 0.00. Therefore, the conclusion is that based on the mean *r*_*WG(J)*_ we reject the null hypothesis that there is no agreement in the ensemble of four vignette hospitals.

Table 6 reports for each scale the sample values for *r*_*WG(J)*_ and *AD*_*M(J)*_ for the four hospitals and the 95^th^ and 5^th^ percentiles of the null distributions for *r*_*WG(J)*_ and *AD*_*M(J)*_ respectively. For both scales, the sample values for *r*_*WG(J)*_ for all four hospitals exceeded the 95^th^ percentile of the null distribution for *r*_*WG(J)*_. Likewise, for both scales, the sample values for *AD*_*M(J)*_ for all four hospitals were smaller than the 5^th^ percentile of the null distribution for *AD*_*M(J)*_. These results indicate that sufficient inter-rater agreement exists for each of the four hospitals to justify the construction of an organizational readiness for implementing change measure from individual-level perceptual data. The organization-level means for the Change Commitment Scale and the Change Efficacy Scale for each hospital are shown in second and third columns of Table 6.

**Table 6:** **Significance tests for the means of *r***_***WG(J) ***_**and *AD***_***M(J) ***_**for each vignette hospital (study three)**

			**Change commitment (CC)**				**Change efficacy (CE)**			
					**Simulation-based**				**Simulation-based**	
			**Sample values**		**Percentiles**		**Sample values**		**Percentiles**	
**Vignette**	**CC mean**	**CE mean**	***r***_***WG(J)***_	***AD***_***M(J)***_	**0.95 *r***_***WG(J)***_	**0.05 *AD***_***M(J)***_	***r***_***WG(J)***_	***AD***_***M(J)***_	**0.95 *r***_***WG(J)***_	**0.05 *AD***_***M(J)***_
Hospital 1	4.33	3.83	0.95	0.54	0.40	1.11	0.89	0.73	0.42	1.10
Hospital 2	3.21	2.33	0.83	0.85	0.42	1.10	0.89	0.69	0.44	1.09
Hospital 3	2.07	2.99	0.82	0.83	0.44	1.10	0.67	0.99	0.43	1.10
Hospital 4	2.07	2.14	0.86	0.71	0.42	1.10	0.83	0.78	0.42	1.10

*Note*: Organizational readiness was manipulated in the vignettes as follows: Hospital 1 high commitment-high efficacy; Hospital 2 high commitment-low efficacy; Hospital 3 low commitment-high efficacy; and Hospital 4 low commitment-low efficacy. The hospital-level means for change commitment (CC) and change efficacy (CE) were consistent with the experimental manipulation. For all four hospitals, the sample values for *r*_*WG(J)*_ exceeded the 95^th^ percentile values of the empirical distributions derived from 100,000 simulated random samples. Likewise, for all four hospitals, the sample values for *AD*_*M(J)*_ were smaller than the 5^th^ percentile values of the empirical distributions derived from 100,000 simulated random samples. Therefore, for each hospital, we reject the null hypothesis of no agreement based on the uniform (rectangular) distribution.

## Study four

### Method

#### Design, participants, and procedure

For study four we used a convenience sample of international non-governmental organizational (INGO) staff (n = 311) from around the world who responded to an online survey about their organizational readiness to implement mobile technology for monitoring and evaluation systems in health programs. All INGOs were based in the United States. Of the study participants, 54.5% were men and 45.5% were women. Three percent of respondents were under 25 years old, 35% were 25 – 35 years old, 35% were 36 – 45 years old, 20% were 45 – 55 years old, and 7% were older than 55. A total of 44% of respondents were based in the United States and 56% were based in other countries (primarily in Africa).

### Measures

The online survey in this study included four items to assess change commitment and five items to assess change efficacy. One of the five change commitment items used in study two and study three (‘We will do whatever it takes to implement this change’) was inadvertently dropped in survey construction.

### Analysis

We conducted a two-factor CFA to assess factor structure, computed alpha coefficients for the resulting scales to assess inter-item consistency, computed ICC(1) and ICC(2) from a one-way random-effects ANOVA to assess the reliability of organization-level means, and tested the statistical significance of *r*_*WG(J)*_ and *AD*_*M(J)*_ to assess the validity of organization-level means. For these assessments, we included only organizations (n = 10) represented by more than five survey respondents.

## Results

The two-factor CFA model converged and demonstrated a good fit when change commitment and change efficacy were allowed to correlate. The comparative fit index (CFI) equaled 0.97, the Tucker-Lewis fit index (TLI) = 0.96, the standard root mean square residual (SRMR) was 0.05 and the root mean square error of approximation (RMSEA) was = 0.08. No post-hoc modifications were necessary given the good fit indicated by the fit indices; residual analysis did not indicate problems. Standardized parameter estimates are provided in Table 7.

**Table 7:** Confirmatory factor analysis of organizational readiness for implementation items (study four)

			CFA* standardized factor loadings	
	**Mean**	**Standard deviation**	**I**	**II**
C1. Are committed to implementing this change	3.07	1.33	0.928 (0.020)	
C2. Are determined to implement this change	2.82	1.29	0.884 (0.025)	
C3. Are motivated to implement this change	2.87	1.45	0.745 (0.044)	
C5. Want to implement this change	3.29	1.32	0.824 (0.033)	
E2. Can manage the politics of implementing this change	2.78	1.07		0.785 (0.043)
E3. Can support people as they adjust to this change	2.77	1.29		0.791 (0.042)
E5. Can coordinate tasks so that implementation goes smoothly	2.71	1.16		0.832 (0.037)
E6. Can keep track of progress in implementing this change	2.97	1.15		0.647 (0.058)
E7. Can handle the challenges might arise in implementing this change	2.91	1.29		0.743 (0.047)

*Notes:* All items began with ‘We’ where ‘We’ referred to the respondent’s global organization. All statements concluded with ‘to mobile technology for MandE systems’.

*Standardized factor loadings for two factor confirmatory factor analysis; Standard Error in parenthesis for CFA: CFI = 0.9768 TLI = 0.955; SRMR = 0.052; RMSEA = 0.08.

Alpha coefficients for the four-item Change Commitment Scale and the five-item Change Efficacy Scale were 0.91 and 0.89 respectively. The correlation between the unit-weighted scale scores was similar to the correlation between the factors (*r* = 0.60, *p <*0.001).

The one-way ANOVA for the Change Commitment Scale yielded an ICC(1) of 0.09 (p < 0.02), and an ICC(2) of 0.56. Using interpretative conventions, the ICC(1) value approximates a ‘medium’ effect size and the ICC(2) value suggests a moderate level of reliability [15,25]. The one-way ANOVA for the Change Efficacy Scale yielded an ICC(1) of 0.02, and an ICC(2) of 0.16 (p <0.30). Using interpretative conventions, the ICC(1) value indicates a ‘small’ effect size and the ICC(2) suggests a low level of reliability. These correlations are lower than those obtained in study three, where we could manipulate and standardize the information that respondents received about the readiness of the four hypothetical hospitals depicted in the vignettes. However, these correlations are close to the values reported for subjective measures in implementation studies in healthcare settings (median = 0.04; interquartile range = 0.01 – 0.06) [26]. Although results would support the aggregation of individual-level data into an organization-level mean for change commitment, they would not support such aggregation for change efficacy.

A different picture emerges from an analysis of the inter-rater agreement statistics. For the Change Commitment Scale, the sample values for *r*_*WG(J)*_ and *AD*_*M(J)*_ for the ensemble of 10 INGOs were 0.82 and 0.72 respectively. For the Change Efficacy Scale, the sample values for *r*_*WG(J)*_ and *AD*_*M(J)*_ for the ensemble of 10 INGOs were 0.82 and 0.76 respectively. Table 8 summarizes the empirical distributions in terms of their means, medians, standard deviations, 5^th^ percentiles, and 95^th^ percentiles. For both scales, the sample values for *r*_*WG(J)*_ for the ensemble of INGOs were larger than the 95^th^ percentile of their corresponding null distributions and the corresponding p-values were almost zero. Likewise, the sample values for *AD*_*M(J)*_ for the ensemble of INGOs were smaller than the 5^th^ percentile of their corresponding null distributions and the corresponding p-values were almost zero. Therefore, for both the *r*_*WG(J)*_ and *AD*_*M(J)*_ indexes, the null hypothesis of no agreement in the ensemble of INGOs was rejected for both the Change Commitment Scale and Change Efficacy Scale. Using interpretive conventions, the values obtained for the ensemble of INGOs suggest ‘strong within-group agreement’ for both change commitment and change efficacy [15,27]. In contrast to the results for ICC(1) and ICC(2), these results would support the aggregation individual-level into organization-level means for *both* change efficacy and change commitment.

**Table 8:** **Significance tests for the means of *r***_***WG(J) ***_**and *AD***_***M(J) ***_**for international non-governmental organizations (study four)**

	**Distribution based on simulation**						
**Variable**	**Mean**	**Median**	**Standard deviation**	**5**^**th **^**percentile**	**95**^**th **^**percentile**	**Sample values**	***p *** **value**
Change commitment (4 items)							
*AD*_*M(J)*_ mean	1.15	1.15	0.05	1.06	1.23	0.72	0.000
*r*_*WG(J)*_ mean	0.20	0.19	0.08	0.07	0.33	0.82	0.000
Change Efficacy (5 items)							
*AD*_*M(J)*_ mean	1.15	1.15	0.05	1.07	1.22	0.76	0.000
*r*_*WG(J)*_ mean	0.21	0.20	0.08	0.08	0.34	0.82	0.000

*Note*: For each of the four statistics (*AD*_*M(J)*_ mean and *r*_*WG(J)*_ mean for each scale), we obtained an empirical distribution based on 100,000 simulated random samples. The distributions are summarized in terms of their means, medians, standard deviations, and 5^th^ and 95^th^ percentiles. Consider the *r*_*WG(J)*_ mean for the four-item Change Commitment scale. Its sample value was 0.82. Simulations under the uniform (rectangular) null distribution indicate that the empirical distribution has a mean of 0.20 and a 95^th^ percentile of 0.33, which is much lower than the observed value of 0.82. Thus, the corresponding *p*-value is 0.00. Therefore, the conclusion is that based on the mean *r*_*WG(J)*_ we reject the null hypothesis that there is no agreement in the ensemble of 10 INGOs.

Table 9 reports the sample values and the 95^th^ and 5^th^ percentiles of the null distributions for *r*_*WG(J)*_ and *AD*_*M(J)*_, respectively, for each scale for the 10 INGOs. In eight of the INGOs, for both scales the sample values for *r*_*WG(J)*_ exceeded the 95^th^ percentile of the null distribution and were smaller than the 5^th^ percentile of the null distribution for *AD*_*M(J)*_. These results indicate sufficient inter-rater agreement for these eight INGOs to justify the construction of organization-level means for change commitment and change efficacy from individual-level data. For INGO 3 and INGO 8, the sample values for *r*_*WG(J)*_ for the two scales did not exceed the 95^th^ percentile of the null distribution nor were the sample values for *AD*_*M(J)*_ for the two scales smaller than the 5^th^ percentile of the null distribution for *AD*_*M(J)*_. These results indicate *insufficient* inter-rater agreement for two INGOs to justify the construction of organization-level means for change commitment and change efficacy from individual-level data.

**Table 9:** **Significance tests for the means of *r***_***WG(J) ***_**and *AD***_***M(J) ***_**for each international non-governmental organization (study four)**

			**Change commitment (CC)**				**Change efficacy (CE)**			
					**Simulation-based**				**Simulation-based**	
			**Sample values**		**Percentiles**		**Sample values**		**Percentiles**	
**Vignette**	**CC mean**	**CE mean**	***r***_***WG(J)***_	***AD***_***M(J)***_	**0.95 *r***_***WG(J)***_	**0.05 *AD***_***M(J)***_	***r***_***WG(J)***_	***AD***_***M(J)***_	**0.95 *r***_***WG(J)***_	**0.05 *AD***_***M(J)***_
INGO 1	4.54	4.00	0.92	0.60	0.62	0.94	0.83	0.72	0.67	0.95
INGO 2	4.30	4.30	0.90	0.67	0.77	0.79	0.87	0.72	0.75	0.86
INGO 3	4.02	4.35	0.57	0.90	0.79	0.74	0.78	0.82	0.81	0.76
INGO 4	3.37	3.41	0.85	0.69	0.76	0.80	0.91	0.60	0.81	0.79
INGO 5	4.03	4.15	0.81	0.76	0.57	1.00	0.82	0.82	0.61	1.00
INGO 6	3.94	4.39	0.96	0.45	0.70	0.85	0.92	0.52	0.70	0.88
INGO 7	4.08	4.37	0.84	0.69	0.42	1.08	0.89	0.65	0.45	1.09
INGO 8	3.48	3.50	0.67	0.94	0.69	0.89	0.55	1.07	0.70	0.92
INGO 9	3.72	3.98	0.88	0.69	0.73	0.84	0.83	0.81	0.60	0.91
INGO 10	3.94	4.20	0.80	0.79	0.61	0.96	0.82	0.80	0.61	0.99

*Note*: CC = change commitment scale (4 items); CE = change efficacy scale (5 items). For eight INGOs, the sample values for *r*_*WG(J)*_ exceed the 95^th^ percentile values of the empirical distributions derived from 100,000 simulated random samples. Likewise, the sample values for *AD*_*M(J)*_ were smaller than the 5^th^ percentile values of the empirical distributions derived from 100,000 simulated random samples. Therefore, these INGOs, we reject the null hypothesis of no agreement based on the uniform (rectangular) distribution. For two INGOs (INGO 3 and INGO 8), sample values for *r*_*WG(J)*_ did not exceed the 95^th^ percentile values of the empirical distributions derived from 100,000 simulated random samples. Likewise, the sample values for *AD*_*M(J)*_ were not smaller than the 5^th^ percentile values of the empirical distributions derived from 100,000 simulated random samples. Therefore, for these two INGOs, we do not reject the null hypothesis of no agreement based on the uniform (rectangular) distribution.

## Discussion

In this article, we provide psychometric evidence in support of a new, brief, theory-based measure of organizational readiness for change, which we call Organizational Readiness for Implementing Change (ORIC). Content adequacy assessment indicated that the items that we developed to measure change commitment and change efficacy reflected the theoretical content of these two facets of organizational readiness and distinguished these two facets from hypothesized determinants of readiness. Exploratory and confirmatory factor analysis in the lab and field study revealed two correlated factors, as expected, with good model fit and high item loadings. Reliability analysis in the lab and field study showed high inter-item consistency for the resulting individual-level scales for change commitment and change efficacy. Inter-rater reliability and inter-rater agreement statistics supported the aggregation of individual level readiness perceptions to the organizational level of analysis.

As expected, the lab study provided stronger evidence than the field study for the reliability and validity of organization-level means as representations of organization-level measures of readiness. In the lab study, we manipulated and standardized the information that study participants received about the organizational readiness of the hospitals depicted in the vignettes to implement meaningful use of electronic health records. In the field study, we made no effort to present study participants with consistent information about their organization’s readiness to use mobile phone technology to monitor and evaluate international health and development programs. Likewise, we made no effort to select international non-governmental organizations that might be expected *a priori* to vary widely in organization readiness. To our knowledge, organizational leaders made no effort to shape organizational members’ perceptions of readiness. Even under these conditions, organizational members exhibited ‘strong agreement’ in their perceptions of organizational readiness, and the overall level of readiness among the set of participating organizations was high.

The discrepant results in the inter-rater reliability statistics [*i.e.*, ICC(1) and ICC(2)] and inter-rater agreement statistics (*r*_*WG(J)*_ and *AD*_*M(J)*_) for change efficacy highlight an important difference in how these two types of statistics measure ‘similarity’ in organizational members’ ratings. Inter-rater reliability indicates the relative consistency (or rank order consistency) in ratings, whereas inter-rater agreement indicate the absolute consensus (or interchangeability) in ratings. LeBreton and colleagues [15] observe that strong levels of inter-rater agreement can be masked by subtle inconsistencies in the rank orders of ratings, especially when the between-unit variance is restricted (*e.g.*, all organizations are rated high or low). For example, two sets of ratings on a seven-point scale (rater one = 7, 6, 6, 7, 7, 6, 6, 7, 7; rater two = 6, 7, 7, 7, 7, 6, 6, 6, 7, 7) would generate a mean *r*_*WG(J)*_ of 0.94 and an ICC(1) of only 0.04. LeBreton *et al.* encourage investigators to examine multiple indicators of inter-rater reliability and inter-rater agreement, but caution that one type of statistic may be more relevant than another depending on the research question. In this case, we were interested in the psychometric question of whether individual perceptual data on readiness could be aggregated to the organization-level of analysis. Our field study results suggested that sufficient consensus existed within the INGOs to measure readiness at the organizational level; however, our results also suggested, for the participating INGOs, between-group variation in change efficacy scores might be insufficient to warrant an organization-level analysis of the determinants or outcomes of this facet of readiness.

Although ORIC shows promise, further psychometric assessment is warranted. Specifically, the measure should be tested for convergent, discriminant, and predictive validity. Convergent validity could be assessed by comparing ORIC to other reliable, valid, but much longer measures, such as the Organizational Readiness for Change Assessment [5] or the Texas Christian University (TCU) Organizational Readiness for Change instrument [28]. Discriminant validity could be assessed by comparing ORIC to measures of constructs related to, yet distinct from, organizational readiness for change (*e.g.*, organizational culture). Finally, predictive validity could be assessed by examining the association of ORIC with hypothesized outcomes of readiness, such as championing change and implementation effectiveness [8]. Assessment of predictive validity is particularly important for determining whether organizational level readiness should be a key priority for leaders of organizational change efforts.

### Limitations

This study had a few limitations. First, one item in the Change Commitment Scale was dropped inadvertently in the field test in study four. Results from our previous three studies suggest that including the item would not have adversely affected the reliability and validity of the scale in study four. Nevertheless, future field studies should check this.

Second, we carried forward to study two one item (*i.e.*, ‘We want to perform this change’) that did not satisfy the conditions for content adequacy in study one. We did so because we believed participants may not have had sufficient semantic context to determine the meaning of ‘want.’ We obtained encouraging results for this item from the exploratory and confirmatory factor analysis in the laboratory study (study two and 3) and the confirmatory factor analysis in the field study (study four). Nevertheless, further testing on this item is warranted.

Third, in study two and study three we asked graduate and undergraduate students to assess organizational readiness as if they were an employee of the hospital described in the vignette. This approach may raise concerns about the validity of these data because the students are not in fact hospital employees. However, we believe this approach is appropriate for our study because it has been used in several previous studies [29,30], the students were enrolled in programs (health policy and management or health behavior) that familiarize students with healthcare settings, and the results of our field test with actual employees (study four) support findings from study two and study three.

Fourth, we could not test for a higher-order organizational readiness for change construct because the structural component of the model would be under-identified with only two factors. Our field study results suggest, however, that constructing a higher-order factor might not be advisable given the moderate correlation of the Change Commitment and Change Efficacy scales and the differences they exhibited in inter-rater reliability. Researchers might wish to retain the scales rather than combine them because they capture related, yet distinct facets of organizational readiness to implement change.

Finally, in study four we did not collect information about the efforts organizational leaders undertook to increase readiness of employees. Such information could have proved useful for assessing why inter-rater reliability for the Change Efficacy Scale did not support aggregation of the individual-level data into an organizational-level mean. For example, it is possible that some individuals were provided more information about the impending change than others, resulting in different views on the organization’s readiness. Finally, because each organization in study four exhibited a high level of readiness, it would be useful to test ORIC in a sample with more variation in readiness between organizations.

## Conclusion

A brief, reliable, and valid measure of organizational readiness for change, such as ORIC, could advance implementation research and practice. For research, such a measure would enable the testing of theories about the determinants and consequences of organizational readiness. Such advancements could lead to answers for a number of important questions: Is organizational readiness for change important for all types of changes and in all types of organizations? Is readiness a necessary, but not sufficient, condition for effective implementation of a change? Is there a readiness threshold that should be met prior to beginning implementation of the change? Do all organizational representatives need to be ready for the change, or is readiness only important for specific groups of individuals? Healthcare leaders could use answers to such questions, combined with ORIC, to assess organizational readiness for change in their own settings. Doing so would be useful for developing implementation strategies and allocating resources for a specific change. Ultimately, valid measurement of organizational readiness for change could reduce the number of health organization change efforts that either do not lead to desired benefits or fail altogether.

## Appendix

### *Reliability: Variance Within and Between Groups*

The intraclass correlation coefficient (ICC) provides an indication of the proportion of group-level variance. ICC(1) equals the correlation between the values of two randomly drawn individuals from a single randomly drawn group. This correlation is commonly interpreted as the proportion of variance in a target variable that is accounted for by group membership. ICC(2) represents the reliability of the group mean scores and varies as a function of ICC(1) and group size, so that large group sizes can result in high ICC(2) values, even if ICC(1) values are low.

Values obtained from a One-Way Analysis of Variance (ANOVA)

$$\mathrm{ICC}\left(1\right)=\frac{\mathrm{MSB}-\mathrm{MSW}}{\mathrm{MSB}+\left(N-1\right)\left(\mathrm{MSB}\right)}$$

$$\mathrm{ICC}\left(2\right)=\frac{\mathrm{MSB}-\mathrm{MSW}}{\mathrm{MSB}}$$

Where *MSB* = Mean Square Between; *MSW* = Mean Square Within; *N* = Number of Individuals in the Group.

### *Cohen et al. r*_*WG(J)*_*and AD*_*M(J)*_

The index r_WG(J)_ compares the observed within-group variances to an expected variance under the null hypothesis of no agreement. For a discrete scale of J parallel items, r_WG(J)_ is defined as:

$$\begin{array}{l} r_{\mathrm{WG}\left(J\right)}=\frac{J\left[1-\left({\bar{s}}^{2}/\sigma^{2}\right)\right]}{J\left[1-\left({\bar{s}}^{2}/\sigma^{2}\right)\right]+{\bar{s}}^{2}/\sigma^{2}}\\ {\sigma^{2}}_{\mathrm{null}}=\left(A^{2}-1\right)/12 \end{array}$$

Where ${\bar{s}}^{2}$ is the average of the observed variances on the *J* items, and *σ*^*2*^ is the variance of a null distribution corresponding to some null response pattern. The most natural candidate to represent non-agreement, which has often been used, is the uniform (rectangular) distribution, accordingly for an item with number of categories which equals A.

The ambiguity in choosing the right null distribution has been recognized as one of the drawbacks of using **r**_**WG(J)**_ and motivated the introduction of alternative indices. Burke et al. (1999) introduced the AD index, defined as follows. For the *j* th item (*j* =1,… *J*):

$$AD_{M\left(j\right)}=\frac{1}{n}\sum_{k=1}^{n}\left|X_{\mathrm{jk}}\_{\bar{X}}_{j}\right|$$

Where *n* is the number of respondents, *X*_*jk*_ is the *k* th respondent’s score on item *j*, and ${\bar{X}}_{j}$ is the mean of the respondent’s score on item *j*. This statistic is also known as the mean absolute deviation.

The index *AD*_*M*(*J*)_ is defined as the average of the *AD*_*M*(*J*)_’s over the *J* items.

$$AD_{M\left(J\right)}=\frac{1}{J}\sum_{j=1}^{J}AD_{M\left(J\right)}$$

## Competing interests

The authors declared that they have no competing interests.

## Authors’ contributions

CS participated in recruiting participants, developing vignettes used in the lab studies, and drafting the manuscript. SJ participated in performing analyses for each study and drafting methods and results Sections. DE performed analyses for studies three and four, and participated in drafting methods and results Sections. KB recruited participants for the field study and participated in drafting the methods section for that study. BW led the study design and participated in recruiting participants and drafting the manuscript. All authors participated in drafting the manuscript and read and approved the final version.

## Supplementary Material

Additional file 1 Organizational Readiness for Implementing Change (ORIC). Click here for file
