# Supplementary material for: Organizational readiness for implementing change: a psychometric assessment of a new measure
Source: Implement Sci. 2014 Jan 10;9:7. doi: 10.1186/1748-5908-9-7 (PMC3904699; doi:10.1186/1748-5908-9-7)
Supplement: Additional file 1 — Organizational Readiness for Implementing Change (ORIC). [file 1748-5908-9-7-S1.doc]

**Additional file 1 Organizational Readiness for Implementing Change (ORIC)**

| 1 | 2 | 3 | 4 | 5 |
| --- | --- | --- | --- | --- |
| Disagree | Somewhat  Disagree | Neither Agree nor Disagree | Somewhat  Agree | Agree |

| 1. People who work here feel confident that the organization can get people invested in implementing this change. | 1 2 3 4 5 |
| --- | --- |
| 1. People who work here are committed to implementing this change. | 1 2 3 4 5 |
| 1. People who work here feel confident that they can keep track of progress in implementing this change. | 1 2 3 4 5 |
| 1. People who work here will do whatever it takes to implement this change. | 1 2 3 4 5 |
| 1. People who work here feel confident that the organization can support people as they adjust to this change. | 1 2 3 4 5 |
| 1. People who work here want to implement this change. | 1 2 3 4 5 |
| 1. People who work here feel confident that they can keep the momentum going in implementing this change. | 1 2 3 4 5 |
| 1. People who work here feel confident that they can handle the challenges that might arise in implementing this change. | 1 2 3 4 5 |
| 1. People who work here are determined to implement this change. | 1 2 3 4 5 |
| 1. People who work here feel confident that they can coordinate tasks so that implementation goes smoothly. | 1 2 3 4 5 |
| 1. People who work here are motivated to implement this change. | 1 2 3 4 5 |
| 1. People who work here feel confident that they can manage the politics of implementing this change. | 1 2 3 4 5 |
